# A novel in vitro model reveals distinctive modulatory roles of *Plasmodium falciparum* and *Plasmodium vivax* on naïve cell-mediated immunity

**DOI:** 10.1186/s12936-017-1781-4

**Published:** 2017-03-27

**Authors:** Setthakit Chitsanoor, Sangdao Somsri, Panyu Panburana, Mathirut Mungthin, Ratawan Ubalee, Maliwan Emyeam, Somchai Jongwutiwes, Jetsumon Sattabongkot, Rachanee Udomsangpetch

**Affiliations:** 1 Department of Pathobiology, Faculty of Science, Mahidol University, Bangkok, Thailand; 2 Graduate Programme in Biomedical Science, Faculty of Allied Health Sciences, Thammasart University, Pathumthani, Thailand; 3 Department of Obstetrics and Gynecology, Faculty of Medicine Ramathibodhi Hospital, Mahidol University, Bangkok, Thailand; 4 Department of Parasitology, Phramongkutklao College of Medicine, Bangkok, 10400 Thailand; 5 Department of Entomology, USAMC Armed Forces Research Institute of Medical Sciences, Bangkok, Thailand; 6 Molecular Biology of Malaria and Opportunistic Parasites Research Unit, Department of Parasitology, Faculty of Medicine, Chulalongkorn University, Bangkok, Thailand; 7 Mahidol Vivax Research Unit, Faculty of Tropical Medicine, Mahidol University, Bangkok, Thailand; 8 Centers for Emerging and Neglected Infectious Diseases, Mahidol University, Bangkok, Thailand; 9 Centers for Research and Innovation, Faculty of Medical Technology, Mahidol University, Nakhon Pathom, Thailand

**Keywords:** Hematopoietic stem cells, Cell mediated immunity, Malaria

## Abstract

**Background:**

To date, human peripheral blood mononuclear cells (PBMCs) have been used mainly in immune stimulation assays and the interpretation of data can be influenced by the previous immunological history of donors and cross reactivity with other infectious agents. Resolving these limitations requires an alternative in vitro model to uncover the primary response profiles.

**Methods:**

A novel in vitro model of mononuclear cells (MNCs) generated from haematopoietic stem cells (HSCs) was developed and these cells were then co-cultured with various antigens from *Plasmodium falciparum* and *Plasmodium vivax* to investigate the response of naïve immune cells to malaria antigens by flow cytometry.

**Results:**

In vitro stimulation of naïve lymphocytes showed that CD4^+^ and CD8^+^ T lymphocytes were significantly reduced (P < 0.01) by exposure to lysates of infected erythrocytes or intact erythrocytes infected with *P. falciparum*. The depletion was associated with the expression of CD95 (Fas receptor) on the surface of T lymphocytes. Maturation of T lymphocytes was affected differently, showing elevated CD3^+^CD4^+^CD8^+^ and CD3^+^CD4^−^CD8^−^ T lymphocytes after stimulation with cell lysates of *P. falciparum* and *P. vivax*, respectively. In addition, antigen presenting monocytes and dendritic cells derived from haematopoietic stem cells showed impaired HLA-DR expression as a consequence of exposure to different species of malaria parasites.

**Conclusion:**

These results suggest that naïve mononuclear cells differentiated in vitro from HSCs could provide a valid model for the assessment of immunity. *P. falciparum* and *P. vivax* malaria parasites could modulate various populations of immune cells starting from newly differentiated mononuclear cells.

**Electronic supplementary material:**

The online version of this article (doi:10.1186/s12936-017-1781-4) contains supplementary material, which is available to authorized users.

## Background

To date, research on adaptive immunity has been restricted to the use of peripheral blood mononuclear cells (PBMCs) as an in vitro model. However, some of these PBMCs are either already committed, or have been primed with unknown epitopes from previously encountered microbes. Given this phenomenon, there might be a number of factors leading to misinterpretation of results, particularly when cross reactivity is concerned [1, 2]. Lymphocytes circulating in the peripheral blood could have already committed to a vast number of non-self-antigens derived mostly from microbial products before being recruited into the experiment. A study in 2004 by Bisset and colleagues [3] supported this theory, and showed that less than half CD4^+^ cells, and less than a quarter CD8^+^ T lymphocytes in the peripheral blood remained naïve. Consequently, several diseases have shown cross reactivity with malaria, i.e. schistosomiasis [4, 5], leishmaniasis, toxoplasmosis, and Chagas’ disease [6].

Resolving these limitations requires an alternative in vitro model such as newly produced immune cells to uncover primary response profiles. However, there is little information on the in vitro application of mononuclear cells (MNCs) derived from haematopoietic stem cells (HSCs) for research in this area. This study establishes a novel in vitro model focusing on the use of naïve T lymphocytes in stimulation assays. Additionally, the system uses newly differentiated immune cells derived from cord blood-HSCs to verify the modulatory roles of *Plasmodium falciparum* and *Plasmodium vivax* in the manipulation of naïve immune cells without the interference of prior donor immunological exposure.

## Methods

### Isolation of cord blood mononuclear cells

Ten samples of umbilical cord blood of normal full-term newborns obtained from Ramathibodi Hospital following informed consent (MURA2014/400 approved by Ethical Committee of Research on Human Beings from Ramathibodi Hospital, Faculty of Medicine, Mahidol University, Bangkok, Thailand) were used to isolate HSCs. Between 50 and 70 ml of cord blood was collected into blood bags containing 30 ml of CPDA-1 anticoagulant [Kawasumi Laboratories (Thailand) Co Ltd]. Cord blood samples were overlaid on Lymphoprep™ solution (Axis Shield PoC, Oslo, Norway) at a 1:1 ratio by volume and centrifuged (Kubota, Tokyo, Japan) at 1200×*g*, 20 °C for 30 min. The interface layer containing the cord blood mononuclear cells (CBMNs) was collected and washed twice with cold PBS buffer containing 2 mM EDTA. Viability of isolated cells was assessed using the trypan blue exclusion method.

### Isolation of CD34^+^ haematopoietic stem cells (HSCs)

HSCs were isolated from CBMNs with a CD34 isolation kit and Mini-MACS columns (Miltenyi Biotech, Germany) according to the manufacturer’s protocol. Briefly, 1 × 10^8^ CBMCs were suspended in 500 µl cold PBS containing 2 mM EDTA and 0.5% fetal calf serum. The cell suspensions were incubated at 4 °C, for 30 min with 100 µl of each FcR blocking reagent and anti-CD34 antibody-conjugated magnetic microbeads (clone: QBEND/10). Subsequently, the cells were washed with cold PBS buffer to remove unbound anti-CD34 antibodies and loaded onto an LS column (Miltenyi Biotech, Germany). The column was rinsed with PBS buffer to remove non-specific cells. The retained CD34^+^ cells were eluted from the column with PBS buffer and centrifuged at 800×*g* for 10 min at 4 °C and the resulting cell pellet was re-suspended in StemlineII^™^ medium, cells were counted and cultured.

### Cultivation and differentiation of HSCs derived from CBMNs (HSC-derived MNCs)

The isolated CD34^+^ haematopoietic stem cells were cultured according to an established protocol [7]. Briefly, 5 × 10^5^ cell/ml of CD34^+^ cells were cultured in 12-well tissue culture plates (Costar^®^, Corning Inc, NY, USA) using StemlineII™ medium (Sigma-Aldrich Corp, MI, USA) supplemented with 50 ng/ml of stem cell factor (Sigma-Aldrich Corp), 10 ng/ml of IL-3 (PeproTech Asia, Rehovot, Israel), 100 µg/ml transferrin (Sigma-Aldrich Corp) and 100 µg/ml of humulin (Sigma-Aldrich Corp). CD34^+^ HSCs were incubated at 37 °C in a humidified atmosphere with 5% CO_2_ and half volumes of medium were replaced with fresh complete medium every three days. Cell number and viability were assessed after five and ten days of cultivation by the trypan blue exclusion method. On day 10 of cultivation, cell surface markers of all mononuclear cells were determined by flow cytometric analysis (FACScan, Becton–Dickinson, Oxford, UK) and cells were morphologically examined after Giemsa staining. Ten days old HSC-derived MNCs were used in co-cultivation with malaria antigens in all assays.

### Cultivation of parasites and antigen preparations

Antigens used in this study were prepared from two sources of human malaria parasites. *Plasmodium falciparum* parasites were obtained from in vitro cultivation of TM267 laboratory strain maintained in group O human erythrocytes from healthy donors by in vitro cultivation, using RPMI-1640 medium (Gibco, Carlsbad, CA. USA) supplemented with 10% human serum. The parasites were incubated at 37 °C in a humidified atmosphere with 5% CO_2_ in air, starting from ring stages until most of the *P. falciparum* parasites entered mature schizont stages.

*Plasmodium vivax* parasites were isolated from blood of 10 *P. vivax*-infected patients (Ethical Approvals WRAIR#1308, MU-IRB 2009/346.2711). Briefly, blood samples were filtered through Plasmodipur^®^ filters (Euro-Diagnostic B.V., The Netherlands) to deplete leukocytes. The resulting *P. vivax*-infected blood were washed twice with McCoy 5A medium (Gibco) by centrifugation at 800×*g*, 4 °C for 5 min and cultured at 5% haematocrit in McCoy’s 5A medium supplemented with 25% human AB-serum. The parasites were kept at 37 °C in a humidified atmosphere with 5% CO_2_ in air for 24–30 h (depending on the age of the starting parasites) until most of the *P. vivax* parasites entered mature schizont stages.

Two types of antigen preparations, intact infected erythrocytes and parasitized cell lysates, were prepared from both species. Schizont stage parasites were isolated by gradient centrifugation at 1200×*g*, 4 °C for 30 min with 60% Percoll^®^ for *P. falciparum* infected erythrocytes, and 45% Percoll^®^ for *P. vivax* infected erythrocytes to enrich the parasite. *Plasmodium falciparum*-infected erythrocytes (PfIEs) and *P. vivax*-infected erythrocytes (PvIEs) in the interface layer between medium and Percoll^®^ were collected and washed twice by centrifugation at 800×*g*, 4 °C for 5 min with cold RPMI-1640. The resulting PfIEs and PvIEs were counted and used to stimulate the HSC-derived MNCs as described below. For the parasite lysates, the same batch pellets of the PfIEs and PvIEs were stored at −70 °C without any cryopreservative agent and twice freeze–thawed for use as whole malaria infected erythrocyte lysates. Uninfected erythrocyte controls were prepared from erythrocytes of healthy donor used in the *P. falciparum* parasite culture by repeating the same protocol as that used for infected erythrocytes as described above.

### Stimulation of HSC-derived MNCs with malaria antigens

On day 10 of cultivation, HSC-derived MNCs from each cord blood sample were individually co-cultured with the different malaria antigens, i.e. intact infected erythrocytes and whole parasite infected erythrocyte lysates from both *P. falciparum* and *P. vivax* (MNCs: antigens = 1:5). Stimulation with intact uninfected erythrocyte (1:5) and whole uninfected erythrocyte lysate (1:5) were used as baseline controls for the leukocyte response. Phytohemagglutinin-A (PHA) (5 µg/ml) was used for validating the activity of T lymphocytes for mitogenic response. On day 2 or day 4 after stimulation, the HSC-derived MNCs were harvested for phenotypic characterization by flow cytometry (FACScan).

### Phenotypic characterization and expression of death receptor (CD95)

Phenotyping of the cells was performed by three-colour flow cytometry (FACScan). Stimulated HSC-derived MNCs (1 × 10^5^ cell) from each condition were labeled with fluorescent, dye-conjugated monoclonal antibodies to define various populations of cells including T lymphocytes (anti-CD3-PECy5, CD4-PE and CD8-FITC), monocytes (anti-CD14-PE and HLA-DR-PECy5), dendritic cells (anti-CD40-FITC, and HLA-DR-PECy5), B lymphocytes (anti-CD19-FITC), NK and NKT cells (anti-CD3-PE and CD56-FITC), HSCs (anti-CD34-PE and Lin-FITC) and expression of death receptor (anti-CD3-PE, anti-CD4-FITC, anti-CD8-FITC and anti-CD95- PECy5) (eBioscience, San Diego, CA, USA; and Miltenyi Biotech, Germany for anti-CD34-PE) (details of antibodies used can be found in Additional file 1). After staining, the cells were washed with PBS pH 7.4 and fixed with 1% paraformaldehyde in PBS. The analysis was performed using the CellQuest software (Becton–Dickinson, San Jose, CA, USA). The mononuclear cells were gated by that excludes those events with low FSC and high SSC, for exclusion of debris and dead cells from the analysis.

### Statistical analyses

The data are represented as fold change of population and analysed by the GraphPad Prism (version 6.0, San Diego, CA, USA) and SPSS (version 18.0, Chicago, IL, USA) programs. Statistical significance was determined by one-way analysis of variance (ANOVA). The Mann–Whitney U test for nonparametric data was used for statistical analysis between the fold changes of population of each condition. The results were considered significant at *P* < 0.05.

$$\left( {{\text{Fold change of population }} = \,\,\frac{\text{Percentage population of each condition}}{\text{Percentage population of media}}} \right)$$

## Results

### Analysis of leukocyte populations obtained from HSC culture

**Fig. 1:**
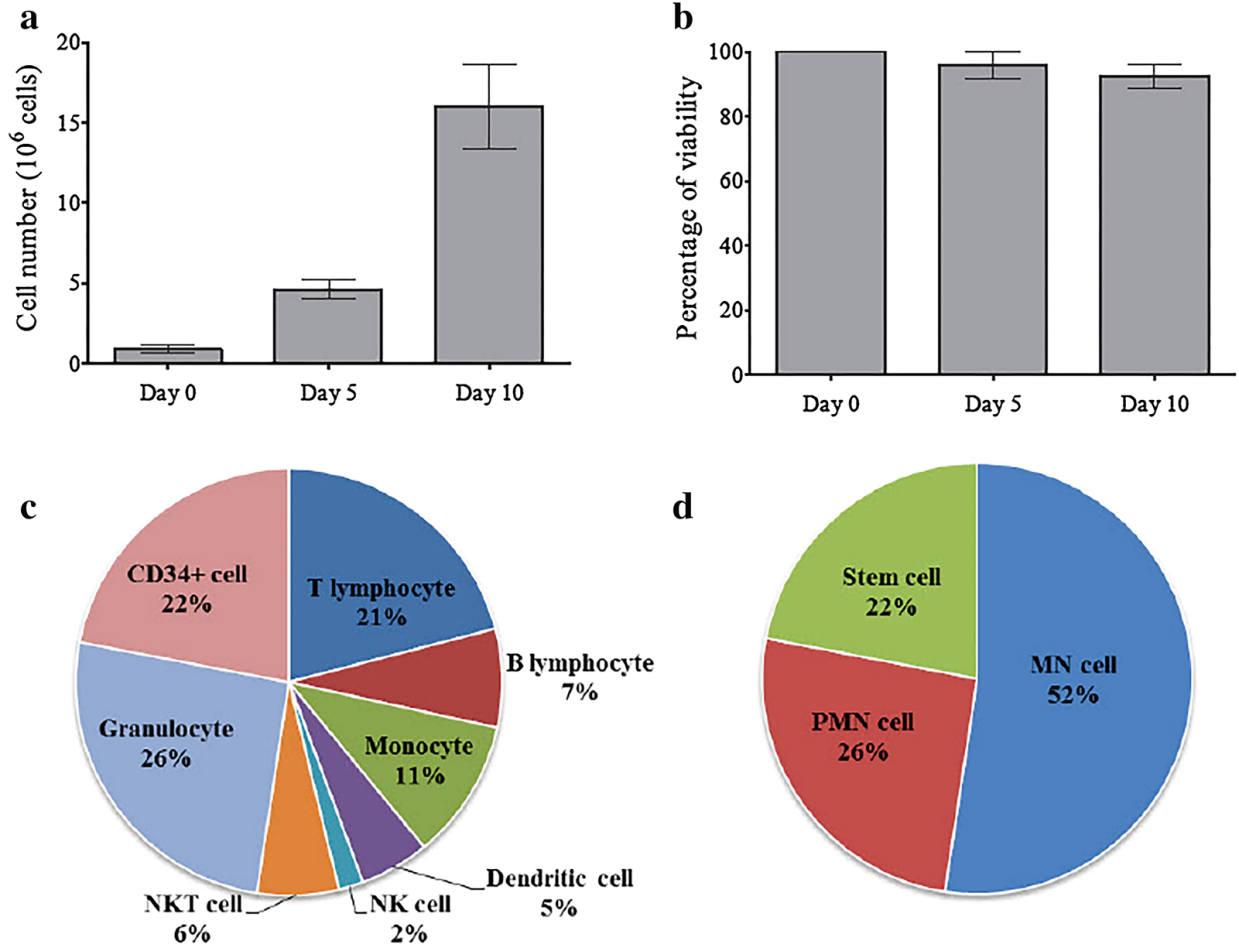
Growth, viability and differentiation of cells from CD34^+^ HSCs in vitro prior exposure to malaria antigens. CD34^+^Lin^−^ cells were isolated using a CD34 isolation kit and cultured in StemlinII™ medium supplemented with suitable growth factors for 10 days. The differentiated leukocytes derived from HSCs were characterized by flow cytometry. The data shown in *bar graphs* represent cell growth (**a**), and viability (**b**) over 10 days (Mean ± SD), with the data being obtained from ten individual cord blood samples. The *pie charts* show the cell population found on day 10 of HSC cultures using flow cytometry characterizations (**c**), and the relative number of cells in the three groups; mononuclear cell, polymorphonuclear cell and haematopoietic stem cell (**d**) (% of total cells). The data are mean values obtained from ten individual cord blood samples

HSC cultures were started with highly purified CD34^+^ Lin^−^ cells (95–98%) and maintained for 10 days. The expansion of total nucleated cells ranged from 5.1 to 8.5-fold on day 5 and 16.6 to 25.15-fold on day 10 after cultivation (Fig. 1a). The viability of the immune cells was more than 90% over the cultivation period (Fig. 1b).

Populations of leukocytes in the HSC cultures were characterized by flow cytometry on day 10 post in vitro cultivation. Several types of immune cell (Fig. 1c) were observed, CD3^+^ T lymphocytes (20.96% ±1.33), B lymphocytes (7.47% ±0.80), monocytes (10.64% ±0.72), dendritic cells (5.28% ±0.72), natural killer cells (1.88% ±0.31), natural killer T cells (6.18% ±1.17) and granulocytes (25.65% ±1.94) (examined by Giemsa staining). The rest were CD34^+^ HSC (21.94% ±2.57) remaining in the culture. Taken together (Fig. 1d), mononuclear cells (52.4% ±2.26) were the majority of immune cells in this HSC culture giving twofold higher numbers than that of the polymorphonuclear cells (25.6% ±1.94).

### Alterations of lymphocyte population by an in vitro exposure to malaria parasites

**Fig. 2:**
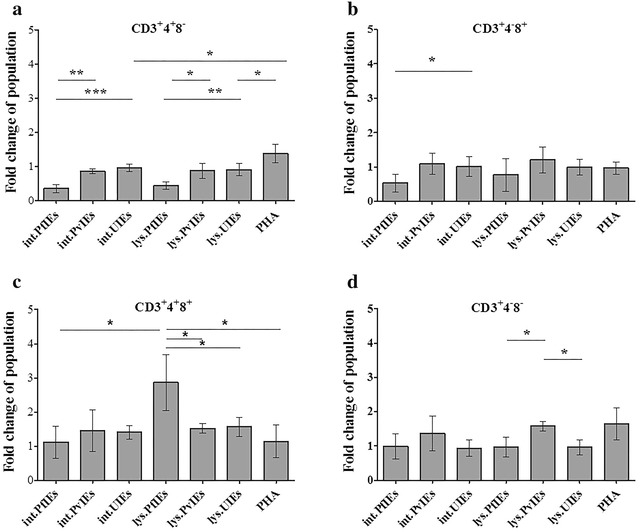
Responses of naïve T lymphocytes to malaria parasites. HSCs-derived mononuclear cells (10 days old) were co-cultured with malaria antigens in various forms and uninfected erythrocyte controls. The *bar graphs* represent the fold changes of T lymphocytes sub-populations after 4 days of co-culture with the malaria antigens. CD3^+^4^+^8^−^ cells (**a**), CD3^+^4^−^8^+^ cells (**b**), CD3^+^4^+^8^+^ cells (**c**), and CD3^+^4^−^8^−^ cells (**d**) with standard deviations. The data are mean values obtained from five individual cord blood samples. *P ≤ 0.05, ***P ≤ 0.001, *int.PfIEs* intact *P. falciparum*-infected erythrocytes, *int.PvIEs* intact *P. vivax*-infected erythrocytes, *int.UIEs* intact uninfected erythrocytes, *lys.PfIEs* cell lysate from *P. falciparum*-infected erythrocytes, *lys.PvIEs* cell lysate from *P. vivax*-infected erythrocytes, *lys.UIEs* cell lysate from uninfected erythrocytes, *PHA* phytohemagglutinin

An inhibitory effect of *Plasmodium* to the newly produced T lymphocytes was observed in the in vitro culture system (Fig. 2). Both intact PfIEs and PfIEs lysates significantly reduced (*P* ≤ 0.001) CD4^+^ T lymphocytes by 0.35 ± 0.12 and 0.45 ± 0.11-fold, respectively (Fig. 2a). Only intact PfIEs could deplete half of CD8^+^ T lymphocytes (*P* ≤ 0.05) (Fig. 2B). Neither antigen from *P. vivax* showed any significant effect on either CD4^+^ or CD8^+^ T lymphocytes compared to their corresponding uninfected erythrocytes control (UIEs). The mitogenic effect of PHA was also observed, showing a 1.4 ± 0.18-fold increases of CD4^+^ T lymphocytes, whereas CD8^+^ T lymphocytes were not affected in the HSC culture (Fig. 2b).

The influence of *Plasmodium* to the transitional stages of T lymphocytes (CD3^+^4^−^8^−^ cells and CD3^+^4^+^8^+^ cells) was monitored in this HSCs culture system. Double positive T lymphocytes (CD3^+^4^+^8^+^ cells) increased 2.5 ± 0.79-fold in the presence of PfIEs lysates (Fig. 2c) whereas double negative T lymphocytes (CD3^+^4^−^8^−^ cells) were elevated 1.6 ± 0.13-fold when exposed to PvIEs lysates compared to UIEs (Fig. 2D) (scatter plots and absolute number of T lymphocyte, can be viewed in Additional files 2 and 3).

### *Plasmodium falciparum* induced expression of CD95 on the surface membrane of T lymphocytes

**Fig. 3:**
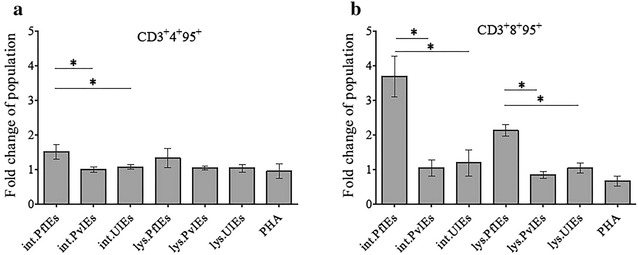
Malaria parasites induce the expression of CD95 on T lymphocytes. HSCs-derived mononuclear cells (10 days old) were co-cultured with malaria antigens in various forms and uninfected erythrocyte controls. The *bar graphs* show the fold changes of CD95^+^ T lymphocyte population after co-culture with the malaria parasite for 2 days, CD3^+^4^+^ (**a**) and CD3^+^8^+^ T lymphocytes (**b**) with standard deviation. The data are mean values obtained from five individual cord blood samples. *P ≤ 0.05

Depletion of T lymphocytes by the malaria parasite was associated with apoptosis through the CD95 pathway. Abundant CD8 T lymphocytes expressing CD95 on their surface were found on day 2 after being exposed to both intact PfIEs (3.69 ± 0.21-fold) and PfIEs lysates (2.15 ± 0.16-fold). CD4 T lymphocytes expressing CD95 were increased slightly in co-cultivation with intact PfIEs (1.52 ± 0.21-fold) compared to all other antigens in this study (Additional file 3). *Plasmodium vivax* did not significantly induce the expression of CD95 on T lymphocytes compared to UIEs (Fig. 3) (scatter plots can be viewed in Additional file 4).

### The diverse effects of *Plasmodium falciparum* and *Plasmodium vivax* on antigen presenting cells

**Fig. 4:**
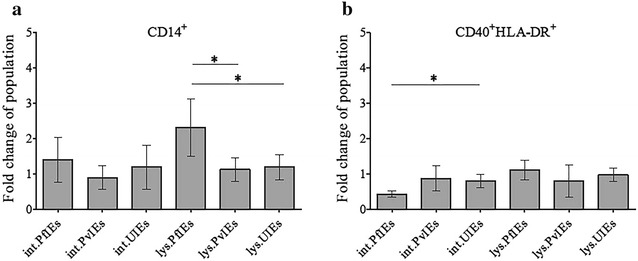
Effects of malaria parasites on the antigen presenting cells. HSCs-derived mononuclear cells (10 days old) were co-cultured with malaria antigens in various forms and uninfected erythrocyte controls. The *bar graphs* show the fold change of antigen presenting cell populations after co-culture with the malaria parasite for 4 days, monocytes (**a**), and dendritic cells (**b**) with standard deviation. The data are mean values obtained from five individual cord blood samples. *P ≤0.05

The effects of malaria parasites on monocytes and dendritic cells were assessed (Fig. 4). PfIEs lysates significantly (*P* ≤ 0.05) increased the number of HSC-derived monocytes by 2.14 ± 0.78-fold when compared to UIEs lysates. Intact PfIEs did not affect the monocyte population but reduced by more than half HSC-derived dendritic cells (0.45 ± 0.09) compared to the intact UIEs (*P* ≤0.05). Neither intact IEs nor the IEs lysates of *P. vivax* showed any effect on monocytes or dendritic cells (scatter plots, can be viewed in Additional file 5).

### Malaria parasites altered the expression of human leucocyte antigen (HLA)-DR on the surface of monocytes and dendritic cells

**Fig. 5:**
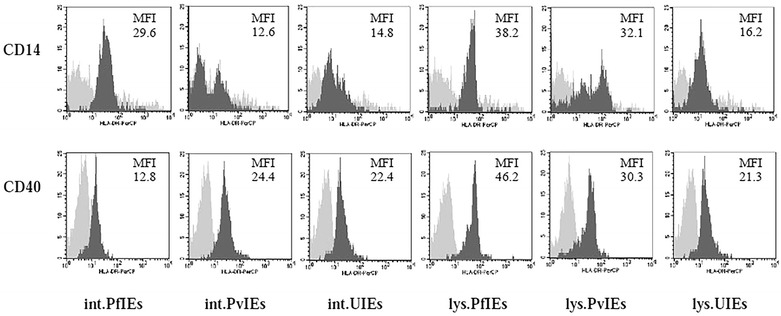
Effects of malaria parasites on HLA-DR expression of antigen presenting cells. HSCs-derived mononuclear cells (10 days old) were co-cultured with malaria antigens in various forms and uninfected erythrocyte controls. The *dark gray histograms* represent the level of HLA-DR expression on the surface of the CD14^+^ cells, and CD40^+^ cells. The *light gray histograms* represent isotype controls after co-culture in the presence of the malaria parasite antigens for 4 days. Data are shown as mean fluorescence intensity (MFI) obtained from a selected cord blood representing the group

To observe any modulation of antigen presenting cells by the malaria parasites, the level of HLA-DR expression by monocytes and dendritic cells was measured. Flow cytometric analysis showed that intact PfIEs increased HLA-DR expression by twofold as shown by increased mean fluorescence intensity (MFI) on the surface of monocytes (CD14^+^ cells). In contrast, intact PvIEs compared with intact UIEs did not show any difference on the overall level of HLA-DR as indicated by the MFI values (12.6 and 14.8, respectively); nonetheless the patterns of HLA-DR expression on CD14^+^ cells under the influence of *P. vivax* antigens were noticeably different compared to that of CD40^+^ cells (Fig. 5). The IEs lysates from both *P. falciparum* (MFI = 38.2) and *P. vivax* (MFI = 32.1) parasites also showed twofold higher HLA-DR expression than that of UIEs lysates (MFI = 16.2).

In dendritic cells, the intact PfIEs (MFI = 12.8) reduced HLA-DR expression by half while intact PvIEs (MFI = 24.4) did not reduce in comparison with intact UIEs (MFI = 22.4). However, PfIEs lysates (MFI = 46.2) and PvIEs lysates (MFI = 30.3) increased the levels of HLA-DR by twofold and 1.5-fold, respectively, on these cells compared to that of UIEs lysates (Fig. 5).

### *Plasmodium falciparum* sustained existence of CD34^+^ HSCs in the culture

**Fig. 6:**
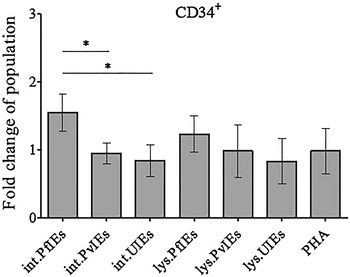
Effects of malaria on the remaining of CD34^+^ cells. HSCs-derived mononuclear cells (10 days old) were co-cultured with malaria antigens in various forms and uninfected erythrocyte controls. The *bar graph* shows the fold changes of CD34^+^ cells remaining in the culture after co-culture with the malaria parasite antigens for 4 days, with standard deviation. The data are mean values obtained from five individual cord blood samples. *P ≤ 0.05

The effect of malaria parasites on the development of HSCs is not well understood. Results obtained from the in vitro model presented in this study showed that intact PfIEs significantly increased the CD34^+^ HSCs remaining in the culture, resulting in a 1.5 ± 0.24-fold higher abundance of these cells when compared to the control (Fig. 6). All other antigens tested did not alter the number of CD34^+^ HSCs in the cultures (scatter plots, can be viewed in Additional file 6).

## Discussion

The development of an in vitro model for HSC-derived MNCs with minimal cytokine supplementation (SCF, IL-3, transferrins and human insulin) [8] generating naïve mononuclear cells is crucial to the study of the response of newly produced immune cells to any agent. Such a system eliminates problems frequently encountered when using PBMCs from donors with various immunological profiles. In the HSC-derived MNC culture, phenotypic markers of mature leukocyte populations were variously expressed on consecutive days as reported previously [7]. On day 10 of cultivation, the expression of all mature leukocytes markers was observed with sufficient cell numbers to perform the experiment. The high percentage of monocytes and dendritic cells obtained under these culture conditions was an additional factor favouring the use of HSC-derived MNCs to study the responses of naïve immune cells to an infection. The activation of newly produced T lymphocytes by malaria antigens was verified by showing up-regulation of an activation marker, CD25 (IL2 receptor) (more view in Additional file 7). This study shows an alternative model to follow the responses of naïve lymphocytes to *P. falciparum* and *P. vivax* malaria.

Immunity to malaria relies on T lymphocytes as the agents for combating malaria parasites in both the pre-erythrocytic and erythrocytic stages [9–11]. However, understanding the mechanism or function of these cells is not yet complete. In the study presented here, *P. falciparum* depleted T lymphocytes in accordance with previous studies [12, 13], although some previous studies have also shown lymphopaenia in *P. vivax* patients [14]. Different mechanisms for the suppression of T lymphocytes by malaria parasites have been proposed, and two possible hypotheses could explain lymphopaenia during acute malaria infection. Firstly the distribution of activated T lymphocyte to other organs such as spleen has been reported in mice [15] although this phenomenon could not be observed in this in vitro system. Secondly, the depletion of T lymphocyte observed in this experiment could occur as a result of the induction of T lymphocyte apoptosis upon malaria parasite infection.

Apoptosis is suggested to be associated with lymphopaenia through various mechanisms during acute infection [16, 17]. The data showed that *P. falciparum* but not *P. vivax* caused the loss of CD4 and CD8 positive T cells and these were associated with the CD95 (Fas)-ligand binding pathway. Our unpublished data indicated that CD95 appeared on lymphocytes after the cells were exposed to *P. falciparum* antigens from day 2 onward and these cells were undetectable on day 4. Therefore CD95 was determined on day 2 whereas other population markers were done on day 4. *Plasmodium falciparum* affected the expression of CD95 of the CD8^+^ subpopulation more than that of CD4^+^ lymphocytes. However, a study in murine malaria showed that depletion of parasite-specific T cells was not via the Fas pathway [18]. Therefore, the present findings confirmed previous report on the difference in immune modulatory roles between human and murine malaria.

In this study, parasite antigens were obtained from different sources, namely *P. falciparum* obtained from in vitro culture and *P. vivax* isolated from endemically infected people with a short period of culture to reach schizont stage. The endogenous RBC membrane components should be the same, and the obvious difference is the membrane associated parasite proteins. Therefore, the difference in the source of parasites should not affect the study outcome, but the difference in species of parasites would play major role in the activation of the naïve immune cells. The results showed the response of HSCs derived T lymphocytes exposed to *P. falciparum* have a higher degree of immune suppression as compared to *P. vivax* in term of amount and polymorphism of proteins [19–21]. The antigenicity of malaria proteins depends on the nature of the proteins and the process of presentation by antigen presenting cells. In this study, particulate infected erythrocytes (intact IEs) of both parasites could alter the population of HLA^−^DR^+^ cells to a greater extent than the parasite lysates. Differential responses of T lymphocytes to different forms of antigen have been documented previously [22, 23].

Malaria infection can suppress erythropoiesis [24, 25], but little is known about the influence on immune cells in the biological niches where development and differentiation take place, i.e. the bone marrow, spleen and lymph nodes. However, a modulatory effect of malaria on the alteration of immune cell populations was demonstrated in this in vitro model. The double-positive developmental stage of T lymphocytes (CD3^+^CD4^+^CD8^+^) was elevated upon exposure to cell lysates of *P. falciparum*. This phenomenon could be associated with the severity of malaria as has been reported for several other infectious diseases, such as HIV [26] and Chagas disease [27], including autoimmune diseases [28] suggesting an immune suppressive role of this cell type [29]. On the other hand, the increment of double-negative developmental stage of T lymphocytes (CD3^+^CD4^−^CD8^−^) when activated with cell lysates of the *P. vivax* malaria parasite, emphasized the difference in the response of newly produced immune cells to the two parasites. Therefore, the consequence of elevated numbers of double-positive and double-negative T lymphocytes upon exposure to proteins extracted from *P. falciparum* and *P. vivax*, respectively, observed here is interesting and deserves further investigation for their roles in immunity to malaria. This study showed for the first time that *P. falciparum* and *P. vivax* differently modulate lymphopoiesis during the immune response to malaria.

Immunity to malaria requires functional co-operation between antigen-presenting cells (macrophages and dendritic cells) and lymphocytes [30, 31], but these antigen-presenting cells can occasionally play a role in severe malaria [32]. In this study, cell lysates from *P. falciparum* raised the proportion of monocytes in the HSC culture which might be associated with the immunopathology of severe malaria. Signs of defective immune priming were also seen in this study, as shown by a decrease in the population of professional antigen presenting cells, dendritic cells, after exposure to *P. falciparum* while the number of these antigen presenting cells was similar to control cultures after exposure to *P. vivax*. In addition, the opposite effects of *P. falciparum* and *P. vivax* on the expression of HLA-DR in monocytes and dendritic cells described here supports previous findings reported by others [33]. Interestingly, the two types of antigen from *P. falciparum* increased expression of HLA-DR on the surface of monocytes which could be an incidence of severe outcome from immune over activation as reported in autoimmune disease patients [34]. In contrast, antigens from *P. vivax* regulated the distinctive pattern of HLA-DR expression on these monocytes which could confer milder symptoms as compared to those caused by *P. falciparum*.

The effect of human malaria on the development of HSCs is not known and whether the prolonged CD34^+^ HSC propagation in vitro was due mainly to activation by *P. falciparum*-infected erythrocytes requires further investigation.

## Conclusion

These finding suggest that immunosuppression by malaria infection possibly takes place at an early stage of lymphocyte maturation. In addition, the use of naïve mononuclear cells derived from HSC as an in vitro model for research in immunity can be easily implemented and would reduce the discrepancy of mononuclear cell quality between different studies.

## Additional files

**Additional file 1.** Fluorescent dye-conjugated monoclonal antibodies used for characterizing of cell phenotypes.

**Additional file 2.** Responses of naïve T lymphocytes to malaria parasites.

**Additional file 3.** Responses of naïve T lymphocytes to malaria parasites (absolute number).

**Additional file 4.** Malaria parasites induced the expression of CD95 on T lymphocytes.

**Additional file 5.** Effects of malaria parasites on the antigen presenting cells.

**Additional file 6.** Effects of malaria on the remaining of CD34^+^ cells.

**Additional file 7.** Activation of naïve T lymphocytes by the lysate of *P. falciparum* infected erythrocytes.
