# Supplementary material for: A novel in vitro model reveals distinctive modulatory roles of Plasmodium falciparum and Plasmodium vivax on naïve cell-mediated immunity
Source: Malar J. 2017 Mar 27;16:131. doi: 10.1186/s12936-017-1781-4 (PMC5368906; doi:10.1186/s12936-017-1781-4)
Supplement: Supplementary file 1 — Additional file 1. Fluorescent dye-conjugated monoclonal antibodies used for characterizing of cell phenotypes. [file 12936_2017_1781_MOESM1_ESM.doc]

**Additional file 1**

**Fluorescent dye-conjugated monoclonal antibodies used for characterizing of cell phenotypes**

| **Monoclonal antibodies** | **Clone** |
| --- | --- |
| **anti-CD3-PECy5** | UCHT1 |
| **anti-CD4-PE** | RPA-T4 |
| **anti-CD8-FITC** | SK1 |
| **anti-CD14-PE**  **anti-CD19-FITC**  **anti-CD56-FITC** | 61D3  HIB19  TULY56 |
| **anti-HLA-DR-PECy5** | LN3 |
| **anti-CD40-FITC** | 5C3 |
| **anti-CD34-PE** | AC136 |
| **anti-Lin-FITC** | Anti-Human CD2 (RPA-2.10)  Anti-Human CD3 (OKT3)  Anti-Human CD14 (61D3)  Anti-Human CD16 (CB16)  Anti-Human CD19 (HIB19)  Anti-Human CD56 (CB56)  Anti-Human CD235a (HIR2) |
| **anti-CD3-PE** | SK7 |
| **anti-CD4-FITC** | RPA-T4 |
| **anti-CD95- PECy5** | DX2 |
|  |  |
