# Supplementary material for: A novel in vitro model reveals distinctive modulatory roles of Plasmodium falciparum and Plasmodium vivax on naïve cell-mediated immunity
Source: Malar J. 2017 Mar 27;16:131. doi: 10.1186/s12936-017-1781-4 (PMC5368906; doi:10.1186/s12936-017-1781-4)
Supplement: Supplementary file 2 — Additional file 2. Responses of naïve T lymphocytes to malaria parasites. [file 12936_2017_1781_MOESM2_ESM.doc]

**Additional file 3**

**Responses of naïve T lymphocytes to malaria parasites**


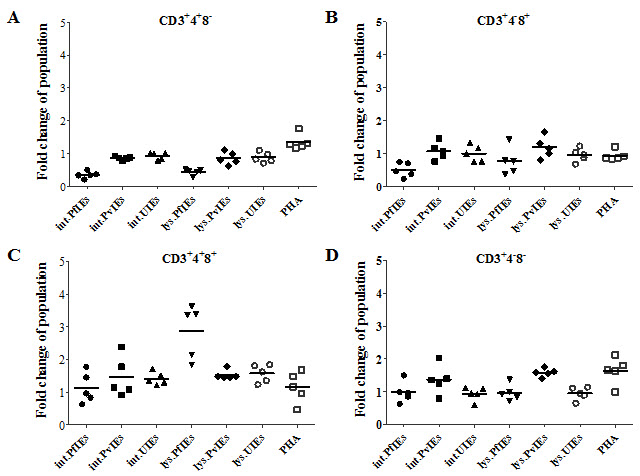


HSCs-derived mononuclear cells (10 days old) were co-cultured with various forms (as described in Materials and Methods) of malaria antigens and uninfected erythrocytes. The scatter plots represent the fold changes of T lymphocyte sub-populations, CD3+4+8- cells (A), CD3+4-8+ cells (B), CD3+4+8+ cells (C), and CD3+4-8- cells (D), after four days of co-cultivation. The data were obtained from 5 cord blood samples.
