# Supplementary material for: A novel in vitro model reveals distinctive modulatory roles of Plasmodium falciparum and Plasmodium vivax on naïve cell-mediated immunity
Source: Malar J. 2017 Mar 27;16:131. doi: 10.1186/s12936-017-1781-4 (PMC5368906; doi:10.1186/s12936-017-1781-4)
Supplement: Supplementary file 3 — Additional file 3. Responses of naïve T lymphocytes to malaria parasites (absolute number). [file 12936_2017_1781_MOESM3_ESM.doc]

**Additional file 4**

**Responses of naïve T lymphocytes to malaria parasites (absolute number)**


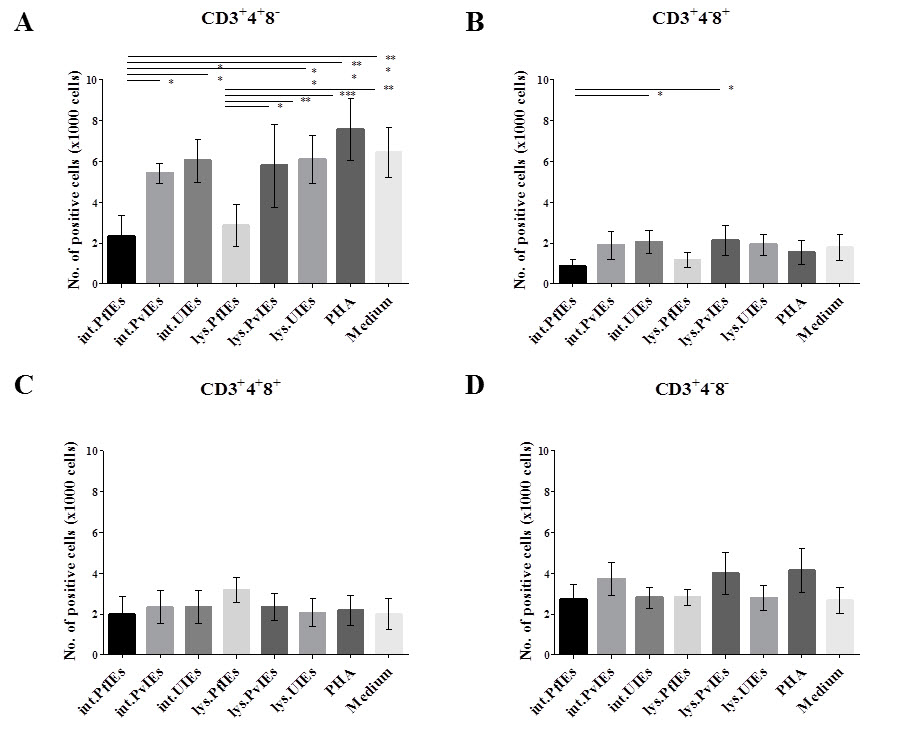


HSCs-derived mononuclear cells (10 days old) were co-cultured with various forms (as described in Materials and Methods) of malaria antigens and uninfected erythrocytes. The bar graphs represent the absolute number of T lymphocytes sub-populations, CD3^+^4^+^8^-^ cells (A), CD3^+^4-8^+^ cells (B), CD3^+^4^+^8^+^ cells (C), and CD3^+^4-8^-^ cells (D), after four days of co-cultivation. The data were obtained from 5 cord blood samples.
