# Supplementary material for: A novel in vitro model reveals distinctive modulatory roles of Plasmodium falciparum and Plasmodium vivax on naïve cell-mediated immunity
Source: Malar J. 2017 Mar 27;16:131. doi: 10.1186/s12936-017-1781-4 (PMC5368906; doi:10.1186/s12936-017-1781-4)
Supplement: Supplementary file 5 — Additional file 5. Effects of malaria parasites on the antigen presenting cells. [file 12936_2017_1781_MOESM5_ESM.doc]

**Additional file 6**

**Effects of malaria parasites on the antigen presenting cells**


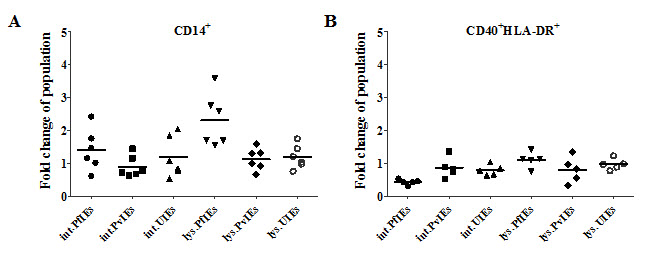


HSCs-derived mononuclear cells (10 days old) were co-cultured with various forms (as described in Materials and Methods) of malaria antigens and uninfected erythrocytes. The scatter plots represent the fold changes of antigen presenting cell populations, CD14^+^ cells (**A**), and CD40^+^HLA-DR^+^ cells (**B**), after 4 days of co-cultivation. The data were obtained from 5 cord blood samples.
