# Supplementary material for: A novel in vitro model reveals distinctive modulatory roles of Plasmodium falciparum and Plasmodium vivax on naïve cell-mediated immunity
Source: Malar J. 2017 Mar 27;16:131. doi: 10.1186/s12936-017-1781-4 (PMC5368906; doi:10.1186/s12936-017-1781-4)
Supplement: Supplementary file 7 — Additional file 7. Activation of naïve T lymphocytes by the lysate of P. falciparum infected erythrocytes. [file 12936_2017_1781_MOESM7_ESM.doc]

**Additional file 2**

**Activation of naïve T lymphocytes by the lysate of *P. falciparum* infected erythrocytes.**


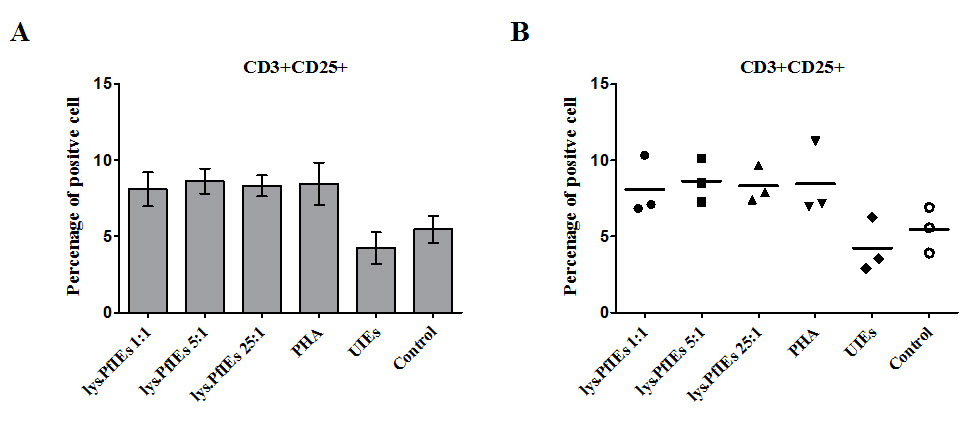


HSCs-derived mononuclear cells (10 days old) were co-cultured with whole cell lysates of *P. falciparum*-infected erythrocytes in various ratios (PfIEs: HSCs = 1:1, 5:1 and 25:1), uninfected erythrocytes (UIEs: HSCs = 5:1) and PHA. The cells cultured in medium alone were used as control without any stimulation (Control). The percentages of CD3+CD25+ cells with standard deviation are presented as bar graphs (A) and scatter plots (B). The data were obtained from 3 cord blood samples.
